# Mitochondrial genotype in vulvar carcinoma - cuckoo in the nest

**DOI:** 10.1186/1423-0127-17-73

**Published:** 2010-09-08

**Authors:** Aleksandra Klemba, Magdalena Kowalewska, Wojciech Kukwa, Katarzyna Tonska, Aleksandra Szybinska, Malgorzata Mossakowska, Anna Scinska, Paweł Golik, Kamil Koper, Jakub Radziszewski, Andrzej Kukwa, Anna M Czarnecka, Ewa Bartnik

**Affiliations:** 1 Institute of Genetics and Biotechnology, Faculty of Biology, University of Warsaw, ul. Pawinskiego 5A, 02-106, Warsaw, Poland; 2 Laboratory of Molecular Oncology, Department of Oncology, Military Institute of the Health Services, ul. Szaserow 128, 04-141 Warsaw, Poland; 3 Maria Skłodowska-Curie Memorial Cancer Centre and Institute of Oncology, Department of Molecular Biology, 5 Roentgena, 02-781 Warsaw, Poland; 4 Department of Otolaryngology, Czerniakowski Hospital, Medical University of Warsaw, 19/25 Stepinska Street, 00-739 Warsaw, Poland; 5 International Institute of Molecular and Cell Biology in Warsaw, 4 Ks. Trojdena Street 02-109 Warsaw, Poland; 6 Institute of Biochemistry and Biophysics, Polish Academy of Sciences, 5A Pawinskiego, 02-106 Warsaw, Poland; 7 Maria Sklodowska-Curie Memorial Cancer Centre and Institute of Oncology, Department of Brachytherapy, 5. Roentgena, 02-781 Warsaw, Poland

## Abstract

Vulvar squamous cell carcinoma (VSCC) is a rare female genital neoplasm. Although numerous molecular changes have been reported in VSCC, biomarkers of clinical relevance are still lacking. On the other hand, there is emerging evidence on the use of mtDNA as a diagnostic tool in oncology. In order to investigate mtDNA status in VSCC patients, haplogroup distribution analysis and D-loop sequencing were performed. The results were compared with available data for the general Polish population, cancer free-centenarians as well as patients with endometrial and head and neck cancer. The obtained data were also compared with the current status of mitochondrial databases. Significant differences in haplogroup distribution between VSCC cohort, general Polish population and cancer-free centenarians cohort were found. Moreover, a correlation between the VSCC patients haplogroup and HPV status was observed. Finally, a specific pattern of mtDNA polymorphisms was found in VSCC. Our results suggest that the mitochondrial genetic background may influence the risk of VSCC occurrence as well as susceptibility to HPV infection.

## Introduction

Vulvar squamous cell carcinoma (VSCC) is a rare female genital neoplasm - it is 2.5% of cancer cases among women, and 5% of all gynecological cancers, which is the 4^th ^ranking cause of morbidity - after breast, cervix and endometrial carcinomas [1]. Today two models of vulvar tumorigenesis are accepted: HPV-associated pathway and HPV-independent pathway. Unfortunately molecular data on VSCC are fragmentary and incoherent [2,3].

Mitochondrial dysfunction has been linked to a wide range of degenerative and metabolic diseases, cancer, and aging with its genome (mtDNA) being considered as "Pandora's box" of pathogenic mutations and polymorphisms [4,5]. MtDNA has a very high mutation rate, which results in three classes of clinically relevant phenotypes. Deleterious germline line mtDNA mutations are linked to mitochondrial diseases, mtDNA polymorphisms are linked to environmental adaptation in human evolution and mtDNA somatic mutations are linked with aging and cancer [6,7]. Mitochondrial defects were first associated with carcinogenesis several decades ago, when Warburg reported 'injury of the respiratory chain' and high glycolysis rate as typical of cancer [8]. Until now the role of mitochondria in neoplasm formation is supported by a growing body of evidence. Today mitochondrial dysfunction does appear to be a factor in cancer etiology [9-12]. Alterations in the mitochondrial genome (mtDNA), including point mutations, deletions, insertions, and genome copy number changes, are believed to be responsible for this phenomenon [13-17]. It is for the fact that mitochondria are pivotal to cell metabolism, but also to regulating cellular signal transduction pathways. It is now postulated that reactive oxygen species (ROS) provide the interface between the mtDNA mutations and cancer progression [18-20].

Mutations have been found in cell lines and tumor-derived samples. Reported mtDNA mutations and polymorphisms were shown to be localized in the entire mitochondrial genome. Nevetheless the highest mutation rate was reported for the displacement loop (D-loop) sequence - the control region of mtDNA, and its two hypervariable regions: HV1 (nucleotides 16024-16383) and HVII (57-372) [10,13,21,22]. High frequency of mtDNA mutations have been reported in variety of cancer types including: bladder, breast, colon, head and neck, liver, lung, prostate, and thyroid cancer. MtDNA alterations are also found in gynecological cancers. Wang *et al. *analyzed 12 mtMSI (mitochondrial instability) regions in cervical, endometrial and ovarian cancer and found that 95.6% of alterations localized in the D-loop [23]. In endometrial carcinoma the occurrence of mtMSI in position 303-315 was shown to correlate with an increased mtDNA content, when normal endometrium and tumor samples were compared [24]. Moreover, somatic mutations in the D-loop, 12 S rRNA and 16 S rRNA sequences were found to be frequent in this type of cancer [25]. The inheritance of mtDNA with haplogroup-D specific polymorphisms localized in the D-loop was shown to increase the risk of endometrial cancer development [26]. The D-loop and cytochrome b gene (*cytB*) were shown to be mutated in 20% of ovarian cancer cases [27]. We have previously shown that as many as 57% of Polish ovarian cancer patients carry somatic mutations in D-loop. Although mutations reported in that study did not correlate with patients' medical history, the mtDNA content in tumor samples was significantly increased in comparison to control - noncancerous ovarian tissue [28].

For several reasons mtDNA seems to be a good target of clinical analyses [9,29,30]. MtDNA is present in thousands of copies within the cell, therefore an infinitesimal amount of the tissue is needed for successful analysis and minimally-invasive procedures may be used to obtain diagnostic material [31,32]. Moreover, mtDNA alterations are easily detectible not only in the tumor sample, but also in body fluids [33]. At the same time, mtDNA mutation and polymorphism analyses are relatively fast and cost-effective [34].

To our best knowledge until today no VSCC patients has been performed. In our opinion, such an experiment fills the gap in gynecological mitochondrial oncology. As a first step to accomplish this goal we screened D-loop of VSCC samples in order to identify somatic mutations and a pattern of inherited polymorphisms Our step was of specificity of haplogroup distribution among SC. Finally, the last step of the study included correlation analysis of molecular characteristics and patients' medical history.

## Materials and methods

### Analyzed cases

#### Cancer cohorts

Tumor samples and control tissue form VSCC cases were obtained in The Maria Sklodowska-Curie Memorial Cancer Centre in Warsaw. The patients were treated for VSCC under standard protocols between 2002 and 2006. Surgery was performed as described previously [35]. All patients enrolled in the study had histopathologically confirmed invasive VSCC of (Table 1). Apart from two patients with a history of ovarian cancer stage III, patients had not previously been treated for any malignancy. Altogether 25 paired tumor and blood samples were investigated. In five cases the tumor margin was also available for analysis. Genomic DNA was isolated from approximately 25 mg of each pulverised with a Microdismembrator II (B Braun Biotech International) sample with a NucleoSpin Tissue kit (Macherey Nagel Inc.) according to the manufacturer's protocol. The presence and genotyping of HPV was performed using Linear Array HPV Detection Kit and Linear Array HPV Genotyping Test (Roche Molecular Systems, Inc) as described previously [36].

**Table 1:** Clinical characteristics of the investigated group of VSCC patients.

Parameter		Number of cases
**Age**	<55	1(4%)
	>55	24(96%)
**Tumor size**	T_1_	4(16%)
	T_2_	17(68%)
	T_3_	3(12%)
**Metastasis**	M_0_	25 (100%)
	M_1_	0 (0%)
**Lymph node status**	N_0_	15 (60%)
	N_1_	4 (16%)
	N_2_	1 (4%)
	N_X_	5 (20%)
HPV infection*	positive	5 (21%)
	16	3(12%)
	58	1(4,%)
	6	1(4%)
	negative	19(79%)

*the HPV status of one of the patients was unknown

The head and neck patients and endometrial adenocarcinoma patients were recruited as described previously [12,34].

#### Control cohorts

The DNA of 84 healthy centenarians was obtained from the Polish Centenarians project DNA-bank localized in The International Institute of Molecular and Cell Biology in Warsaw. All centenarians had negative cancer medical history and negative family history of cancer [37].

General Polish population data was obtained from our previous analysis [38] and the analysis performed by Malyarchuk *et al. *[39].

All investigated populations share the same ethnicity, nationality, parentage, descent and reside in Poland. This study did not include any patients of Asian, African-American or Jewish origin [34].

The mtDNA research project was approved by the local Ethics Committee at the Medical University of Warsaw, Poland (KB-0/6/2007 to AMC, and KB-0/7/2007 to WK). The VSCC project was approved by the local Ethics Committee of the Cancer Center at the Institute of Oncology (44/2002 to JR). The centenarians' project was approved by local Ethics Committee of the Central Clinical Hospital of the Military Medical Academy (currently Military Medical Institute) in Warsaw. The Centenarians Database was registered at the Bureau of the Inspector General for the Protection of Personal Data in May 1999.

### Polymorphisms and mutations analysis

The mtDNA sequences were obtained from tumor, blood and normal tissue (tumor margin) and aligned to the revised Cambridge Reference Sequence (rCRS) and sequence variants were recorded [40,41]. Germline (inherited) polymorphism was defined as a difference between normal tissue sequence and rCRS. Polymorphism is present both in normal and tumor tissues of a particular patient. Whenever the difference between mtDNA sequences obtained from tumor sample and normal tissue (blood or margin) occured, it was defined as a somatic mtDNA mutation. All described mtDNA alterations were plotted against data from mtDB (35) and MITOPAP databases [41,42].

### D-loop sequence analysis

D-loop region (mtDNA 16024-576) was amplified with three overlapping pairs of primers (Table 2). Each of the forward primers contained FM13 (TGTAAAACGACGGCCATG) sequence at the 5' site, and each of the reverse primers contained RM13 (CAGAGGACAGCTATGACC) tail at the 5' site. PCR was carried out in a MJ Research Dyad dual block thermocycler (Bio-Rad) with the following cycling conditions: initial incubation 3'at 95°C, followed by 30 cycles: (30" 95°C, 30" 55°C, 1' 72) with a final extension step for 7' at 72°C. Two microlitres of the PCR product were analyzed on an ethidium bromide-stained 1.5% agarose gel (30' 80 V) for quantification purposes. Sequencing reactions were carried out at Oligo.pl^©^.

**Table 2:** Primers used in D-loop sequencing.

Primer name	Primer sequence	Position in mtDNA
**FM13.D1F**	AATGGGCCTGTCCTTGTAG	15879-15897
**RM13.D1R**	AACGTGTGGGCTATTTAGGC	16545- 16526
**FM13.D2F**	CGACATCTGGTTCCTACTTC	16495-16514
**RM13.D2R**	GGGTTTGGTTGGTCCGGG	559-542
**FM13.D3F**	CGCTTCTGGCCACAGCAC	315-332
**RM13.D3R**	GGTGTGGCTAGGCTAAGC	803-786

The quality of the obtained chromatograms was assessed in FinchTV^® ^software version 1.4.0 (Geospiza Inc., USA). All sequences were analyzed and corrected manually when necessary. Subsequently chromatograms were imported into Sequencher^® ^4.1.4 software (Gene Codec Corporation, Ann Arbor, MI USA) and D-loop contigs were assembled (minimal overlap 20 bp, 85% of identity).

### Haplotyping by RFLP and D-loop analysis

The patterns of specific polymorphisms in mtDNA determine classes of related genotypes, referred to as haplogroups (H, I, J, K, T, U, V, W, X; Table 3). The mtDNA fragments containing polymorphic sites characteristic for specific haplogroups were amplified by PCR according to the following cycling conditions: initial incubation for 3' at 95°C, followed by 35 cycles (30" 95°C, 30" 55°C, 1' 72) with a final extension step for 7' at 72°C. One microlitre of PCR product was analyzed on an ethidium bromide-stained 1.5% agarose gel (30' 80 V) and then digested by appropriate restriction enzymes (overnight, 37°C). The digestion products were analyzed on an ethidium bromide-stained 2.5% agarose gel (75', 60 V). Tree diagram was used to facilitate haplogroup analysis [43]. In addition to canonical loci (Table 3 - positions 1-14), new RFLP reactions were designed for the project and additional positions in mtDNA were also investigated (Table 3 - positions 14-22) [41,43-45]. In addition to coding sequence analysis the D-loop sequence was also analyzed in order to establish haplotype of each patient. The haplogroup assignment was done as previously published [11,28]. Finally it was also validated with mtDNA search engine [46].

**Table 3:** RFLP haplogroup analysis.

	Haplogroup	Polymorphism	Enzyme	Primer F	Primer R	Primer F - sequence	Primer R - sequence	PCR product	RFLP DNA fragments if this haplogroup	RFLP DNA fragments if not this haplogroup
**1**	**H**	**C7028T**	Alu I	6730F	7398R	CTATGATATCAATTGGCTTCC	GGCATCCATATAGTCACTCC	669	**342, 158, 139, 30**	342, 188, 139
**2**	**U/K**	**A12308G**	Hinf1	11902F	12328R	GCTAGTCCACGTTCTCCT	TTTGGAGTTGCACCAAGAATT	427	**162, 158, 59, 48**	221, 158, 48
**3**	**K**	**G9055A**	HaeII	8563F	9231R	ACAATCCTAGGCCTACCCG	GATAGGCATGTGATTGGTGG	669	**669**	494, 175
**4**	**I**	**G16398A**	BamHI	15879F	16545R	AATGGGCCTGTCCTTGTAG	AACGTGTGGGCTATTTAGGC	667	**511, 156**	667
**5**	**I**	**T10031C**	Alu I	9821F	10516R	ACTTCACGTCATTATTGGCTC	ATGGAGATGGTAATTGCTAG	696	**283, 209, 204**	413, 283
**6**	**I**	**G8251A**	AvaII	7960F	8641R	ATTATTCCTAGAACCAGGCG	TGATGAGATATTTGGAGGTGG	682	**392, 290**	682
**7**	**I**	**A4529T**	HaeII	4184F	4869R	TCCTACCACTCACCCTAGC	GTCATGTGAGAAGAAGCA	686	**686**	350, 336
**8**	**W**	**G8994A**	HaeIII	8563F	9231R	ACAATCCTAGGCCTACCCG	GATAGGCATGTGATTGGTGG	669	**266, 205, 187, 11**	266, 205, 156, 31, 11
**9**	**T**	**A15607G**	AluI	15372F	16067R	TAGGAATCACCTCCCATTCC	GTCAATACTTGGGTGGTACC	696	**236, 218, 170, 72**	406, 218, 72
**10**	**T**	**G13368A**	BamHI	12951F	13614F	CGCTAATCCAAGCCTCACC	TATTCGAGTGCTATAGGCGC	691	**691**	416, 248
**11**	**J**	**G13708A**	BstNI	13568F	14276R	TTACTCTCATCGCTACCTCC	GGTTGATTCGGGAGGATCC	709	**709**	571, 138
**12**	**J**	**C16069T**	Hinf1	15879F	16545R	AATGGGCCTGTCCTTGTAG	AACGTGTGGGCTATTTAGGC	667	**480, 122, 65**	602, 65
**13**	**V**	**G4580A**	NlaIII	4184F	4869R	TCCTACCACTCACCCTAGC	GTCATGTGAGAAGAAGCA	686	**684, 2**	397, 287, 2
**14**	**X**	**G1719A**	DdeI	1138F	1801R	GAACACTACGAGCCACAGC	TCATCTTTCCCTTGCGGTAC	664	**188, 187, 134, 111, 30, 14**	188, 187, 111, 86, 48, 30, 14
**15**	**W1**	**A11947G**	BsmFI	11765F	12108R	GCACTCACAGTCGCATCATAA	TTGAGGGATAGGAGGAGAATG	343	**196, 149**	343
**16**	**M**	**C10400T**	AluI	10381F*	10671R	AAAAAGGATTAGACTGAGCTGA	CGGCAAAGACTAGTATGGCAA	318	**201, 172, 18**	219, 72
**17**	**J, T, L2, H**	**T4216C**	NlaIII	4142F	4379R	GATTCCGCTACGACCAACTC	GCACGGAGAATTTTGGATTC	197	**160, 78**	197
**18**	**HV**	**C14766T**	MseI	14642F	14968R	CCCACACTCAACAGAAACAAA	AGCGGATGATTCAGCCATAA	346	**203, 142, 4**	203, 125, 17, 4
**19**	**U/K**	**A1811G**	PsiI	1623F	1909R	GCACCCAACTTACACTTAGGA	TTTCGGGGGTCTTAGCTTT	287	**287**	188, 101
**20**	**K**	**A10550G**	NlaIII	10387F	10761R	GATTAGACTGAACCGAATTGG	CGGCAAAGACTAGTATGGCAA	285	**164, 121**	285
**21**	**T**	**A4917G**	BfaI	4865F	5192R	ATGACAAAAACTAGCCCCCA	AGGGTGGATGGAATTAAGGGT	348	**298, 39, 11**	337, 11
**22**	**X**	**T6221C**	Mnl I	5881F	6254R*	GCCATTTTACCTCACCCCCACTGATGTTCG	TATAGCAGATGCGAGCAGGAGTAGGAGATAGGGA	374	**131, 115, 108, 20**	115, 108, 106, 25, 20

Restriction enzymes and primers used indicated. * mismatched primers.

### Statistical analysis

Two tailed non-directional Fisher-Irwin (Fisher's exact test) was used for statistical analysis [47]. Statistical analysis was performed with PAST (PAlaeontological Statistics) software *ver. 1.34 *(Øyvind Hammer, D.A.T. Harper and P.D. Ryan, 2005) and Analyse-it for Microsoft Excel General & Clinical Laboratory modules Version 1.73 (Analyse-it Software, Ltd. Copyright © 1997-2005). The difference was considered statistically significant if *p *< 0.05. To confirm the result of Fisher's test - Yates's chi and un-corrected chi squared test ('*N *- 1' chi squared test) were used to give relatively low Type I error in the case of a small number of cases analyzed. The statistics was performed as previously recommended by Campbell [48]. To further calculate the significance of specific polymorphisms as factors of favorable outcomes (odds ratio, relative risk, difference in proportions, absolute and relative reduction in risk) and the effectiveness of a diagnostic criterion (number needed to diagnose, specificity, positive and negative predictive values, positive and negative likelihood ratios, diagnostic and error odds ratios) additional analyses have been performed. These parameters, as well as the confidence intervals for the estimated parameters were computed by general methods [49,50].

## Results

### Haplogroup analysis

**Figure 1:**
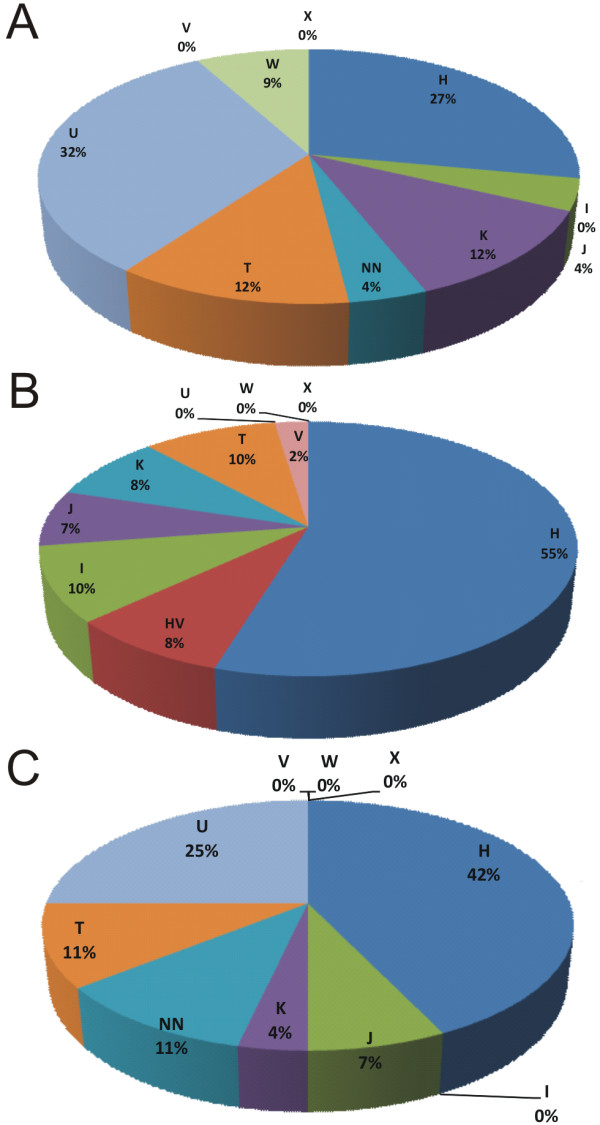
**Haplogroup distribution in the studied group of VSCC patients (A) and haplogroup distribution in control populations: cancer free centenarians - (B) and head and neck cancer patients (C)**.

Haplogroup distribution in the group of 25 VSCC patients is shown in Figure 1A: seven patients (27%) belong to haplogroup H, eight (32%) to haplogroup U, three (12%) to K and T, two (9%) to W, and one (4%) to haplogroup J. One patient (4%) could not be classified to any investigated European haplogroup, due to unspecific polymorphisms pattern. Haplogroup assessment with the mtDNA search engine [46] revealed that this patient probably harbors mtDNA polymorphisms characteristic for East Asian haplogroup Y1b, which suggested other than Polish descent [51]. No patients were found to be classified haplogroup I, V and × members. As expected [44,52], no patient showed a positive RFLP pattern for haplogroup M.

In order to verify haplogroup distribution in the VSCC cohort differs from that of the healthy Polish population and whether any similarities to the other cancer comparisons were made: 1. with general Polish population (PP and NP) [38,39], 2. with cancer-free centenarians (CENT), 3. with head and neck cancer (HNC) 4. with endometrial adenocarcinoma cohort (EA) [53] (Tables 4 and 5). First of all, the underrepresentation of haplogroup H in the VSCC cohort was found. In other words haplogroup H was overrepresented in healthy individuals (cancer free centenarians - CENT). Moreover, in the comparison with the combined general Polish population (COMB), a trend towards haplogroup U overrepresentation was also noticed. A trend for haplogroup K overrepresentation was also found. When further comparison of super-haplogroup UK (encompassing haplogroup U1-U7 and haplogroup K) frequency was made (Table 6), its overrepresentation in VSCC patients became highly significant (44 vs. 19%, p = 0.009). Finally, a comparison with cancer free centenarians shows a trend of overrepresentation of haplogroup W in the VSCC cohort (Table 4, Figure 1A and 1B). As expected, the distribution of haplogroups in VSCC is similar to other studied cancer groups (HNC and EA).

**Table 4:** Analysis of the specificity of haplogroup distribution in VSCC cohort.

				*1*	*2*	*3*	*4*	*5*	*6*
	*Positive*	*Negative*	*% positive*	*p vs PP*	*p vs NP*	*p vs COMB*	*p vs CENT*	*p vs HNC*	*p vs EA*
**H**	7	18	28%	0.502	0.102	0.152	**0.023 U**	0.39	0.173
**I**	0	25	0%	1	1	1	0.194	1	1
**J**	1	24	4%	0.697	0.7	0.712	1	1	0.191
**K**	3	22	12%	0.381	0.067	0.092	0.693	0.333	1
**NN**	1	24	4%	1	0.547	0.552	ND	0.613	0.61
**T**	3	22	12%	1	1	1	0.712	1	0.668
**U**	8	17	32%	0.3	0.052	0.104	**0.01 O**	0.761	0.764
**V**	0	25	0%	0.368	0.385	0.389	1	1	1
**W**	2	23	8%	0.147	0.254	0.209	0.051	0.218	1
**X**	0	25	0%	1	1	1	1	1	1
**Total**	**25**								

Haplogroup distribution in VSCC patients cohort was were compared with 1) the general Polish population (p vs PP) [38], 2) population from the Northern Poland (p vs NP) [39], 3) those two populations combined (p vs COMB), 4) cancer free centenarians cohort (p vs CENT), 5) head and neck tumors patients cohort (p vs HNC), and 6) endometrial adenocarcinoma patients cohort (p vs EA) [11]. % positive - percentage of patients carrying particular haplotype in VSCC cohort. Significant differences - bolded: O - overrepresented in VSCC cohort, U - underrepresented in VSCC cohort.

**Table 5:** Analysis of the specificity of haplogroup distribution HNC cohort.

		*1*	*2*	*3*	*4*	*6*
	*% positive*	*p vs PP*	*p vs NP*	*p vs COMB*	*p vs CENT*	*p vs EA*
**H**	0.43	0.67	0.847	1	0.286	0.015
**I**	0	1	1	1	0.197	1
**J**	0.07	1	1	1	1	0.243
**K**	0.04	1	1	1	0.677	0.184
**NN**	0.11	0.11	0.065	0.065	ND	1
**T**	0.11	1	1	1	1	1
**U**	0.25	0.62	0.29	0.31	0.053	1
**V**	0	0.22	0.629	0.389	1	1
**W**	0	1	0.614	1	1	0.227
**X**	0	1	1	1	1	1

Haplogroup distribution in VSCC patients cohort was were compared with 1) the general Polish population (p vs PP) [38], 2) population from the Northern Poland (p vs NP) [39], 3) those two populations combined (p vs COMB), 4) cancer free centenarians cohort (p vs CENT), 5) head and neck tumors patients cohort (p vs HNC), and 6) endometrial adenocarcinoma patients cohort (p vs EA) [11]. % positive - percentage of patients carrying particular haplotype in VSCC cohort. Significant differences - bolded: O - overrepresented in VSCC cohort, U - underrepresented in VSCC cohort.

**Table 6:** Comparison of UK super-haplogroup frequency between VSCC and 1) the general Polish population [38], 2) population from Northern Poland [39], and 3) those two populations combined.

	*1*	*2*	*3*
	*p vs PP*	*p vs NP*	*p vs COMB*
**Fisher two-tailed**	0.099	**0.009**	**0.000**
**Fisher L**	0.973	0.998	1.000
**Fisher R**	0.070	**0.006**	**0.000**

Significant differences - bolded.

The underrepresentation of haplogroup H in the VSCC cohort was particularly interesting. Haplogroup H is marked by T7028C polymorphism. Therefore, if haplogroup H is underrepresented in the VSCC cohort, 7028T polymorphism is overrepresented. This suggests that a positive RFLP test (7028T) may indicate an increased susceptibility to VSCC. In order to check the properties of this RFLP test additional analyses were performed. VSCC harbour 7028C in 28% of cases, while centenarians carry single nucleotide polymorphism (SNP) 7028C in 55% cases. The difference is significant at p = 0.023 with Fisher's exact test. This significance is further confirmed with Yates corrected chi square test, with χ^2^= 4.50 and p = 0.034, but also with 'N-1' chi square test, with χ^2 ^= 'N-1' and chi square = 5.47 and p = 0.02. he 7028T test has Odds Ratio (OR) and Diagnostic Odds Ratio = 3.11 and indicates Relative Risk (RR) = 2.43 in comparison to cancer free centenarians. Moreover, this test seems cost-effective as Number Needed to Diagnose (NND) is 3.73. The Positive Predictive Value (PPV) of the test is 0.32 at sensitivity 0.72, while the negative predictive value (NPV) is 0.67 and relative risk reduction (RRR) is 0.59.

## Sequencing data analysis

### Germ-line polymorphisms in the mtDNA D-loop region in VSCC

**Figure 2:**
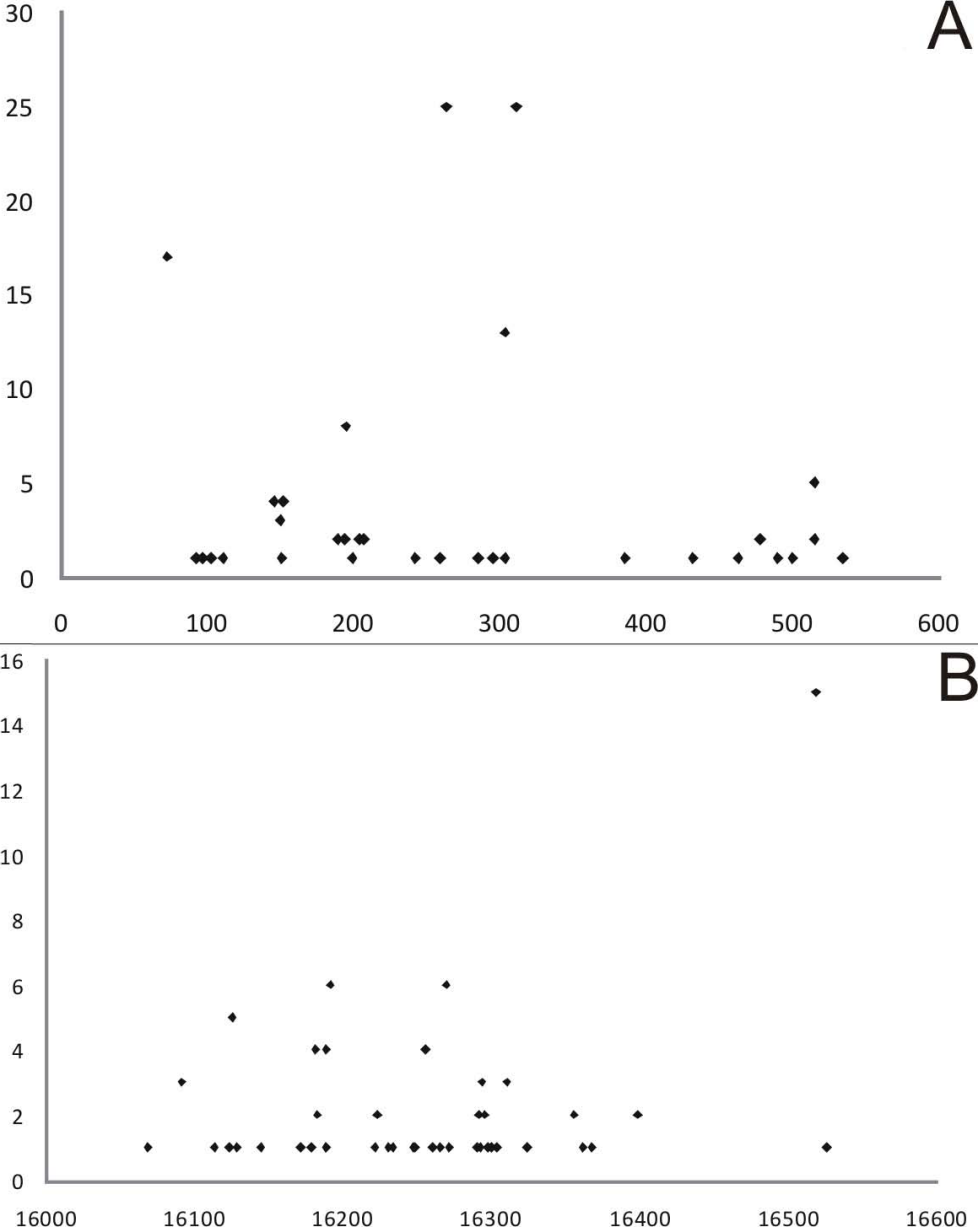
**D-loop polymorphism distribution in the studied group of VSCC patients 0 - 700 mtDNA bp (A) 16000 - 16659 bp mtDNA (B)**. × axis - mtDNA position (bp) - polymorphism location. Y axis - number of cases found in this study - polymorphism number.

Altogether 25 paired mtDNA D-loop sequences from both tumor and blood samples were sequenced. The results are summarized in Table 7. The group of VSCC patients is characterized by 78 germ-line polymorphism (differences haplogroup H - rCRS) [40]. In particular 19 out of 78 polymorphisms are generally uncommon [42] and one polymorphism had not been reported previously (C498delC). Polymorphisms were predominantly found in mtDNA hypervariable regions HV1 (16024-16383) and HV2 (57-333) - 42/78 (54%) and 23/78 (29.5%) polymorphisms, respectively (Table 7, Figure 2A and 2B). Eight (10%) SNPs were localized in HV3 (438-574). The analysis of the control region haplogroup specific loci revealed significant overabundance of certain polymorphisms in the investigated VSCC group of patients. These overrepresented polymorphisms include C16192T, C16256T and C16270T, all being specific for haplogroup U, which is in accordance with the trend observed by haplogroup comparison (Table 6 and Figure 1). The other overrepresented SNPs include 195C, 259G, 477C, 498delC, 533C, 16092G, 16189A, 16248T, 16272G, 16362C. The 16223T sequence variant, characteristic for haplogroups I, W and X, was underrepresented in the VSCC cohort, again reflecting the haplogroup distribution trend. All those results suggest that specific polymorphisms may be found in VSCC. These polymorphisms include not only haplogroup-specific polymorphisms (Figure 1.), but also D-loop polymorphisms (Table 7). No somatic mutations were found.

**Table 7:** Germ-line polymorphisms in the D-loop sequence of VSCC patients.

*mtDNA* *position* *(rCRS)*	*Polymorphism*	*Case No*	*No*	*A/G/C/T/del frequency mtDB*	*Tissue where sequence was found*	*P-value*	*Region/population where sequence variant predominantly found*
**73**	A > G	15,16,17,25, 26,27,29,30, 38,40,41,42, 45,46,49,50,51	17	309/**1555**/1/0/0	*pancreatic cancer*, *thyroid tumor*, *oral cancer *aging brains, POLG/PEO & control muscle,	0.056	Very common
**93**	A > G	27	1	1824/**41**/0/0/0	*ovarian cancer*	0.432	Japan Finland, Italy India
**97**	G > A	33	1	Nd	Polymorphism	-	Nd
**103**	GCC > DEL GCC	33	1	Nd	*oral cancer*	-	Nd
**111**	A > C	33	1	Nd	Polymorhism	-	Nd
**146**	T > C	15,33,49,51	4	1/0/**190**/1674/0	*prostate tumor, ovarian carcinoma *elderly fibroblasts, aging/AD brains, POLG/PEO & control muscle,	0.316	Africa, Japan, Taiwan, Finland, Italy, Spain, Algerian Jew, India, Polynesia, Caucasi
**150**	C > T	16,41,50	3	0/2/1616/**247**/0	*lung tumor*, *thyroid tumor *elderly fibroblasts/leukocytes	1	China, Japan, Berbers, Italy
**151**	C > T	16	1	0/0/1817/**48**/0	Polymorphism	0.484	Japan, Finland, Sweden, India
**152**	T > C	13,26,33,49	4	0/0/**396**/1469/0	*pancreatic cancer, ovarian carcinoma, oral cancer* aging brains, elderly fibroblasts,	0.803	Africa, China, Japan, American, Finland, Italy
**189**	A > G	26,29	2	1782/**75**/8/0/0	*prostate tumor *elderly muscle, POLG/PEO muscle & fibroblasts, aging brains	0.271	Japan, Finland, India
**194**	C > T	26,29	2	0/0/1797/**68**/0	POLG/PEO muscle	0.236	Japan, Indian
**195**	**T > C**	**26,29,39,42, 44, 46,50**	**8**	**11/0/280/1574/0**	***lung-cancer cells***, ***thyroid tumor***, ***oral cancer*** **elderly fibroblasts, aging/AD brains**,	**0.042 O**	Africa, Japan, American, Finland, Italy, Caucasian
**199**	T > C	29	1	0/0/**121**/1741	*ovarian cancer*, POLG/MNGIE muscle	1.000	Japan, Finland, India
**204**	T > C	26,29	2	0/0/**123**/1741/0	*oral cancer*, *prostate tumor*	0.679	Japan, Finland, India
**207**	G > A	26,29	2	**123**/1742/0/0/0	*oral cancer, prostate tumor,thyroid tumor*	0.679	Japan, Finland, India
**242**	C > T	15	1	0/0/1854/**11/0**	POLG/PEO muscle	0.148	*Extremely rare*, American, Finland
**259**	**A > G**	**40**	**1**	**1866/1/0/0/0**	***Liver cancer***	**0.026 O**	*Extremely rare*, Thaiwan
**263**	A > G	13,15,16,17, 18,22,25,26, 27,28,29,30, 33,34,38,39, 40,41,42,44,45,46,49,50,51	25	6/**1861**/0/0/0	*oral cancer *POLG/MNGIE muscle,	1.000	Africa, Japan, China, Australia, American, Finland, India
**285**	C > T	46	1	0/0/1860/**7**/0	elderly fibroblasts	0.101	*Extremely rare*, Italy, India
**295**	C > T	15	1	4/0/1788/**75/0**	*Glioblastoma*,POLG/MNGIE muscle	1.000	American, Finland, India, Caucasian
**303**	C7 > C8 ins	16,17,26,28,29,30,38,39,40,41,42,45,51	13	-	*multiple tumor types*		Africa, Japan, Taiwan, Finland, Italy, Spain, India, Polynesia, Caucasian, Ashkenazi Jew, American, Australia
**303**	C7 > C9 Ins	18	1	-	*multiple tumor types*		Africa, Japan, Taiwan, Finland, Italy, Spain, India, Polynesia, Caucasian, Ashkenazi Jew, American, Australia
**311**	C5 > C6 Ins	13,15,16,17, 18,22,25,26, 27,28,29,30, 33,34,38,39,40,41,42,44,45,46,49,50,51	25	-	*multiple tumor types*		Africa, Japan, Taiwan, Finland, Italy, Spain, India, Polynesia, Caucasian, Ashkenazi Jew, American, Australia
**385**	A > G	46	1	1861/**5**/0/1/0	Twinkle/PEO frontal cortex	0.077	*Extremely rare*, Koraga
**431**	C > T	39	1	1/0/1852/**14**/0	*ovarian cancer*	0.181	*Rare*,Japan
**462**	C > T	15	1	0/0/2073/**71/0**	*thyroid tumor*	0.572	American, Finland, India, Caucasian
**477**	**T > C**	**13,34**	**2**	**0/1/19/2124/0**	***ovarian tumor*** **AD brains**	**0.023 O**	*Rare*, American, European
**489**	**T > C**	**15**	**1**	**0/0/777/1367**	***ovarian carcinoma prostate tumor***	**0.000 U**	Africa, China, Japan, American, Finland, Italy, India
498*	**C > del C**	**49**	**1**	**0/0/2143/1/0**		**0.023 O**	*Extremely rare*
**499**	G > A	50	1	**38**/2106/0/0/0	*thyroid tumor *prostate tumors	0.366	Japan
**514**	CA5 > CA4	25,39,41,44, 46	5	-	*ovarian carcinoma, thyroid tumors*, *gastric carcinomas*	-	Nd
**514**	CA5 > CA6	29,49,	2	-	*ovarian carcinoma & control tissue*, *thyroid tumors, breast tumors*	-	Nd
**533**	**A > G**	**16**	**1**	**2142/2/0/0/0**	**Polymorphism**	**0.034 O**	*Extremely rare*, Japan, Sicily
**16069**	C > T	15	1	0/0/1793/**73**/0	*oral cancer*	1.000	American, Caucasian, Finland, Italy
**16092**	**T > C**	**15,16,39**	**3**	**0/0/22/1845/0**	***oral cancer***	**0.004 O**	*Extremely rare*, Japan, India
**16114**	C > A	45	1	**8**/0/1858/1/0	Polymorphism	0.113	*Extremely rare*, Japan, Finland
**16124**	T > C	29	1	0/0/**9**/1858	Polymorphism	0.125	*Extremely rare*,South Africa, Korea
**16126**	T > C	15,17,25,42,51	5	0/0/**166**/1701/0	*oral cancer*	0.069	China, Japan, American, Finland, Italy, India
**16129**	G > A	33	1	**304**/1554/9/0/0	*oral cancer*	0.164	Arica, Japan, America, Italy
**16145**	G > A	15	1	**50**/1817/0/0/0	*oral cancer*	0.497	Japan, American, Finland, Italy
**16172**	T > C	15	1	0/0/**150**/1717/0	*head/neck tumor* *back-mutation, oral cancer *MNGIE tissues,	0.716	Japan, Morocco, Finland, Europe
**16179**	C > T	50	1	0/0/1862/**5**/0	Polymorphism	0.077	Asia, Austrlia
**16182**	A > C	22,41,42,46	4	1706/1/**117**/2/41	*prostate tumor*	0.071	China, Japan, Finland, Taiwan Aborigine, India
**16183**	A > C	41,42	2	1541/12/**237**/0/77	lung tumor back-mutation, prostate tumor	0.761	China, Japan, Finland, Taiwan Aborigine, India
**16189**	**T > A**	**39**	**1**	**0/0/522/1345/0**	**Polymorphism**	**0.013 O**	*Extremely rare*
**16189**	T > CC	22	1	-	*endometrial tumor, familial breast* *cancer*		
**16189**	T > C	41,45,42,46	4	0/0/**522**/1345/0	*endometrial tumor, familial breast* *caner*	0.261	
**16192**	**C > T**	**16,27,29,38**, **40,45**	**6**	**0/0/1854/13/0**	***oral cancer***	**0.000 O**	Japan, Finland, Italy
**16222**	C > T	15	1	0/0/1852/**15**/0	*oral cancer*	0.192	American,
**16223**	**C > T**	**26,29**	**2**	**0/0/992/875/0**	***oral cancer***	**0.000 U**	Africa, Japan, China, Australia, India, Finland, Ashkenazi Jews
**16224**	T > C	33,49	2	0/0/**107**/1760/0	*oral cancer*	0.652	Japan, American, Finland, Ashkenazi Jews
**16231**	T > C	51	1	0/0/**11**/1856/0	*oral cancer*	0.148	*Extremely rare*, Japan
**16234**	C > T	26	1	0/0/1800/**67**/0	*oral cancer*	0.602	China, Japan, Ashkenazi Jews
**16248**	**C > T**	**51**	**1**	**0/0/1865/2/0**	***ovarian tumor***	**0.039 O**	*Extremely rare*, Spain, India
**16249**	T > C	46	1	0/0/**87**/1780/0	*prostate tumor*	1.000	Ethiopia, Japan, Italy
**16256**	**C > T**	**27,38,40,45**	**4**	**0/0/1838/29/0**	***ovarian tumor***	**0.001 O**	Japan, Finland, India
**16261**	C > T	15	1	0/0/1756/**111**/0	*oral cancer*	1.000	Japan,Taiwan Aborigine, American, India
**16266**	C > T	52	1	9/4/1820/**34**/0	*oral cancer*	0.375	Japan, India
**16270**	**C > T**	**16,27,38,40,41,45**	**6**	**0/0/1802/65/0**	***oral cancer***	**0.000 O**	Finland, Italy, India
**16272**	**A > G**	**25**	**1**	**1865/2/0/0/0**	**Polymorphism**	**0.039 O**	*Extremely rare *Taiwan Aborigine, India
**16291**	C > T	38	1	0/3/1816/**48**/0		0.483	Japan, Italy
**16292**	C > T	26,29	2	0/2/1801/**64**/0	*breast, ovarian, head/neck tumor*, *oral tumor*	0.216	Japan, Finland, Italy
**16293**	A > G	39	1	1848/**17**/2/0/0	glioblastoma	0.214	*Rare*, Italy, India
**16294**	C > T	17,25,45	3	0/0/1760/**107**/0	-	1.000	Japan, American, Finland, Italy
**16296**	C > T	17,25	2	0/0/1823/**44**/0	*head/neck tumor*	0.122	American, Italy, India
**16298**	T > C	42	1	0/0/**169**/1698/0	*oral cancer* prostate tumor	0.721	China, Japan, Finland, American
**16300**	A > G	29	1	1861/**6**/0/0/0	*head/neck tumor*	0.089	*Extremely rare*, Japan
**16301**	C > T	39	1	0/0/1860/**7**/0	*esophageal, breast & prostate tumors*	0.101	*Extremely rare *Melanesia
**16304**	T > C	25	1	0/0/**140**/1727/0	*esophageal, breast & prostate tumors*	1.000	Japan, American, Finland, Italy
**16311**	T > C	33,39,49	3	0/0/**340**/1526/0	*oral cancer*	0.602	Africa, Japan, China, American, Finland, Italy, India
**16324**	T > C	17	1	0/0/**49**/1818/0	*esophageal cancer*	0.490	Japan, Taiwan Aborigine
**16325**	T > C	29	1	0/0/**47**/1820/0	Polymorphism	0.476	Japan, India
**16356**	T > C	22,50,	2	0/0/**27**/1840/0	*oral cancer*	0.055	India, Australian, Aboriginee
**16362**	**T > C**	**46**	**1**	**1/0/444/1422**	***oral cancer***	**0.016 O**	India, Australian, Aboriginee
**16368**	T > C	49	1	0/0/**15**/1852/0	*gastric carcinoma*	0.192	*Extremely rare*, Japan, Italy
**16399**	A > G	38,40,	2	1828/**38**/0/0/0	*gastric carcinoma* *oral cancer*	0.096	Japan, Italy
**16519**	T > C	13,17,18,22,25,26,28,29,33,34,41,42,49,50,51	15	0/0/**1115**/752/0	*pancreatic, cancer,oral cancer, gastric, lung, ovarian* *tumor*	1.000	Africa, Japan, Caucasian, China, American, Finland, Italy, India
**16526**	G > A	45	1	19/1848/0/0/0	Polymorphism	0.235	Finland, Onge
		**total**	**225**				

Unless stated otherwise, the data are from MITOMAP [63] and mtDB [42] databases.

*-previously not reported, O-overrepresented, U-underrepresented; over and under-represented polymorphisms bolded.

### Germ-line polymorphisms in the mtDNA coding region in VSCC cohort

The segments adjacent to the D-loop sequence (15879-803, Table 8) were also analyzed. Two polymorphisms were found within the 12 S rRNA gene, with the frequency characteristic for the world population [42].

**Table 8:** Polymorphisms found in the coding region of mtDNA of VSCC patients.

*mtDNA position* *(rCRS)*	*Polymorphism*	*Case No*	*No*	*A/G/C/T/del frequency mtDB*	*P-value*	*Region/population where sequence variant predominantly found*
**709** **12 S rRNA**	G > A	17,25,26,29,33, 41,42,46	8	**444**/2260/0	0.053	Africa, native Americans, Japan, American, Finland, Italy, India
**750** **12 S rRNA**	A > G	13,15,16,17,18,22,25,26,27,28,29,30,33,34,38,39,40,41,42,44,45,46,49,50,51	25	22/**2682**/0/0	1.000	CRS is a rare variant; G is very common in the whole world, A/G is consensus mutation

### mtDNA D-loop sequence analysis in the tumor margin

In the case of five patients (13, 38, 45, 46, 49), tumor margin samples were also available. Comparison of the D-loop sequence from the sample triplets (tumor, blood and margin) revealed no difference between these tissues in all the investigated cases, again indicating the presence of inherited polymorphisms and lack of somatic mutations.

#### Correlation with clinical parameters

As the polymorphisms are inherited phenomena, no correlation with TNM or clinical stage was performed. However, an interesting co-incidence of HPV infection with the specific mitochondrial haplogroup was observed. Four out of five patients with HPV infection carried haplogroup H. When taking into account only high risk HPV-16, all infected patients belonged to this haplogroup. The correlation between these two parameters was shown to be statistically significant (Table 9). This correlation is further supported by the very similar haplogroup distribution found in the head and neck tumors, that are also HPV-dependent (Figure 2C).

**Table 9:** HPV infection status and haplogroup co-incidence in VSCC patients.

		HPV total				HPV 16			
		+	-	*total*	*p-value*	+	-	*total*	*p-value*
**Haplogroup H**	+	4	3	7	p = 0.014	3	4	7	p = 0.017
	-	1	16	17		0	17	17	
	*total*	5	19	24*		3	21	24*	

*- 24 patients were included in the analysis, as in one case the HPV unknown.

## Discussion

Although several molecular alterations on the genomic, genetic, epigenetic and protein level have been described in VSCC, no useful molecular markers with possible clinical application have been established so far [28]. Analysis of the mitochondrial genome may provide novel cancer biomarkers for the risk assessment, diagnosis and prognosis. Mitochondrial DNA polymorphisms and/or mutations are commonly used as molecular markers in a wide range of disciplines, from human migration and population studies, to metabolic diseases, and may also prove useful in VSCC management [54]. mtDNA analysis is attractive due to its relatively low cost and lack of requirement for sophisticated technical support, thus making it accessible for most hospital laboratories [10,55,56].

To the best knowledge of the authors, this the first report on mtDNA status in VSCC patients. The analysis of haplogroup distribution revealed a trend toward haplogroup U and K overrepresentation, haplogroup H underrepresentation as well as super-haplogroup UK overabundance in a group of VSCC patients in comparison with a large Polish control population, VSCC. These data suggest that mitochondrial genetic background may be related to an increased risk of VSCC incidence. The inheritance of haplogroup U was previously associated with increased risk of prostate cancer and renal cancer in white North American individuals [32,57]. Haplogroup K was also shown to increase the risk of breast cancer development among European-American women [58], whereas haplogroup U decreased the risk of this condition. The UK super-haplogroup has been hypothesized to confer less coupling efficiency of ETC, thus generating less ROS than other haplogroups (H, J and T), and would be expected rather to have a protective effect on cell damage. This hypothesis is supported by association with increased brain pH in patients with psychiatric disorders [59] and decreased risk of Parkinson disease in individuals with this haplogroup [60]. However, the functional role of genetic background conferred by UK super-haplogroup in cancer has not been investigated. To fully assess the value of the observed effect in the Polish population, a comparison of the observed haplogroup frequencies with mtDNA variant distribution in the healthy population with matched sex, age and ethnicity as well as the study on a larger patient cohort is needed [34,61].

The second part of our study was focused on the D-loop sequence analysis in VSCC, as it has been previously found to be altered in gynecological cancers [10,13,15,23,32]. mtDNA polymorphisms characteristic for the VSCC patients were identified. Some of those polymorphisms are located in functionally important regions of the D-loop. The mitochondrial control region contains several elements essential for mtDNA replication and transcription. Sequence variants in this region may affect binding affinity of trans-acting factors, resulting in an altered rate of mitochondrial replication and transcription. Both increased and decreased mtDNA copy number have been found in several types of cancer [21]. Moreover, the polycytidine tract at nucleotide positions 303-315 was highly polymorphic in all investigated VSCC patients. This region is part of CSB II (299-315) that is essential for proper primer formation [62]. In previous studies an *in vitro *assay approach has shown that premature termination of transcription occurs immediately downstream to CSBII at positions 300-282, which is the site of the RNA-DNA transition point. Premature termination was abolished in 319-289 mutants, whereas mutation at nucleotide positions 304-300 showed a drastic decrease of this process [62], indicating an important role of these sequence variants in the transcription/replication switch. Moreover, the screen of human mtDNA control sequence variants revealed that the variant defining haplogroup J - C295T increased TFAM protein binding and *in vitro *L-strand transcription. Cybrid-based analysis of this variant demonstrated an increased copy number (2 times, J >H), but there was no difference in transcript levels. This fact provides strong support for functional importance of mtDNA control region variants, additionally underlying the role of haplogroup-defining mutations within the D-loop [29]. This variant was also found in the VC patient population. The other polymorphisms localized within mtTFAM binding sites found in the investigated cohort are G259A, C285T, C431T, A533G and mtMSI at np 514-523. These variants may possibly affect DNA-protein interactions. SNP 259A is extremely rare [42]. According to data in MITOMAP, it was also found in liver cancer, but this observation was unpublished [63].

Other highly polymorphic loci in our VSCC group of patients are at nucleotide positions 514-523. These polymorphisms are carried by seven VSCC patients. The clinical significance of 514-523 polymorphisms as biomarkers was proved in a study on breast cancer: patients with multiple alleles of (CA) polymorphism (showing heteroplasmy) had poorer survival in comparison to patients with a homoplasmic (CA) variant, thus suggesting the role of this dinucleotide repeat in cancer formation [64]. Moreover, our analysis of the D-loop sequence also revealed that three polymorphisms characteristic for haplogroup U (C16192T, C16256T and C16270T) as well asC16223C polymorphism are significantly overrepresented in the investigated group of patients. They are all located within HV1 region. 16192T is found within microsatellite (16184-16193) and was found to be a hot spot for mutations in several types of cancer [23]. This sequence variant may be important as it co-localizes with the 3' end of TAS and with the 7 S DNA binding site that are hypothesized to play a role in mtDNA synthesis [65]. Moreover, the T16189C variant was associated with an increased susceptibility to endometrial carcinoma in Chinese women [66]. The 16223C SNP was also found at a significantly higher frequency in VSCC patients compared with the normal population, which is in similar to the data obtained in our laboratory for endometrial carcinoma [11]. Furthermore, the T195C variant, overabundant in VSCC patients, was previously reported in lung [33] and thyroid cancer [67], as well as in brains of Alzheimer patients [68]. The exact role of T195C, C16223C, C16256T and C16270T is unknown. It should be noted that, as there are only a few observations for each polymorphism, the power to detect whether these SNPs play a role in VSCC incidence is relatively low and research performed on larger number of patients is needed. However, the obtained results strongly suggest a role for all these polymorphisms in conferring genetic susceptibility to VSCC development; and they appear to be attractive biomarkers of VSCC risk assessment.

In VSCC patients no somatic mutations were detected, which is in contradiction with several previous studies for other cancer types [21]. It may be claimed that blood is not a proper reference tissue, as mitochondrial mutations were also found in body fluids [33]. However, this should not be the case in the investigated cohort of patients, as none of the patients showed distant metastases, and vulvar tumor expansion occurs through the lymphatic system [69]. In addition, D-loop sequence from tumor margin also showed no alterations. Lack of somatic mutations in cancer samples is similar to previous study finding only polymorphisms in the samples of colon cancer [70]. There are also data proving the hypothesis that mtDNA polymorphisms are significantly associated with cancer development [26,71]. Several studies report low mutation rate and an abundance of mtDNA polymorphisms in cancer samples [72]. Moreover, recent re-analyses of somatic tumor-specific alterations revealed that most of them are in fact sequence variants commonly found in the general population [73-77]. Furthermore, a critical review of mtDNA mutations reported in numerous papers suggested that many of them were actually artifacts generated by methodological and technical errors, and that a lot of somatic and "novel" germline alterations seem thus to result simply from improper sample management and data analysis [78].

Another important topic of mtDNA studies in gynecological oncology is correlation of clinic-pathological characteristics with molecular markers. Due to the fact that only the germline polymorphisms were found, no correlation with TNM parameters was assessed. Interestingly, all three patients with the high risk HPV subtype 16 had haplogroup H. When analyzing all infected patients, the statistically significant overrepresentation of haplogroup H was still present. There is no literature data on any correlation between the mitochondrial haplogroup and HPV infection, but some information from research on cervical cancer is available, however, only papers focusing on the influence of HPV on mtDNA damage have been published to date. In the study of Sharma *et al*., a correlation between mtDNA mutations and HPV infection was found [79]. However, it cannot be concluded from the report whether the mutations were somatic or germline, as DNA sequences from tumors of patients were compared to the MITOMAP database, not to any control tissue. In another study on cervical cancer, HPV positive tumors were found to contain an increased number of somatic mutations (in comparison to precancerous stages). On the other hand, haplogroup H turned out to be highly protective against AIDS progression [80], implying that mitochondrial background might play a role in the course of virus-related diseases. The result obtained for the VSCC cohort suggests that haplotype may confer genetic susceptibility towards HPV infection. To confirm the relation of these two parameters, a study on a larger group of patients is necessary.

Our data obtained of VSCC strongly suggest the role of mtDNA polymorphisms in modifying the risk of this type of cancer incidence and opens new perspectives in search for novel VSCC molecular markers. Therefore, it seems plausible that mtDNA analysis (possibly combined with other molecular markers) may help to identify individuals at risk of developing VSCC. The mitochondrial genetic background is also likely to play a role in predisposition to HPV infection, creating a hope for the establishment of a novel molecular diagnostic tool. However, to fully evaluate the prognostic potential of the discovered alterations, investigation of a more representative patient group is necessary. Such research should include, in addition to the information on mtDNA status, also data about other molecular alterations found within the tumor, thus allowing a broader perspective for an assessment of the role of mtDNA in tumorigenesis. It should be noted that our study includes only the data obtained from the sequencing of the D-loop region. In order to obtain a complete picture of a potential role of mtDNA in the formation and expansion of this cancer, it would be necessary to perform whole mitochondrial genome sequencing, including other cancer-related tissues (from recurrences, distant metastases or margin). Such experiments should also allow to further estimate the prognostic value of data based on the D-loop sequence itself, and possibly reveal some other potential risk factors, as it was the case for G10398A in breast cancer among African American-women [81]. Moreover, as the data were compared with general mitochondrial databases [42,63], the idea of comparison with the mtDNA sequences (D-loop and coding region) from healthy Polish subjects would also be valuable. Although acquiring a representative set of such data is labour-intensive, in further perspective it seems to be worth the effort.

## Conclusions

Mitochondrial research may enable establishment of bio-markers allowing the identification of individuals at high risk for vulvar cancer and to develop screening approaches and early diagnosis tools. Moreover, analysis of mtDNA polymorphism (and possibly also mutational) pattern may be used to select population at increased risk of cancer development, who should be enrolled in extensive screening program. Molecular assessment of mitochondrial abnormalities of cancer cells could represent a promising tool not only for prognosis and early diagnosis of neoplasia, but possibly also during the follow-up of cancer patients. If cost-effectiveness of molecular techniques is considered. PCR seems to be the optimal method for outpatient treatment regimens. Additionally, the application of PCR-coupled with gel electrophoresis or DNA sequencing may be used for rapid analysis of multiple samples in hospital laboratories. In the clinical context, the high frequency of mitochondrial genome instability, in combination with PCR-based assays of high sensitivity, may be of potential usefulness. In future studies related to cancer epidemiology, the significance of a particular mtDNA polymorphism(s) should be analyzed together with other polymorphisms in mtDNA and in nuclear DNA.

## Competing interests

The authors declare that they have no competing interests.

## Authors' contributions

AMC, AlK, MK, WK, PG and EB made substantial contributions to the conception and design of the research, AlK, AMC, EB, PG and MK were involved in drafting the manuscript, AlK, AMC, KT, KK, MK and AlS performed the research, MK, JR, AnK, AnS and MM collected the samples. AMC has given final approval of the version to be published. All authors read and approved the final manuscript.
